# 5-Dodecanolide interferes with biofilm formation and reduces the virulence of Methicillin-resistant *Staphylococcus aureus* (MRSA) through up regulation of *agr* system

**DOI:** 10.1038/s41598-019-50207-y

**Published:** 2019-09-24

**Authors:** Alaguvel Valliammai, Sivasamy Sethupathy, Arumugam Priya, Anthonymuthu Selvaraj, James Prabhanand Bhaskar, Venkateswaran Krishnan, Shunmugiah Karutha Pandian

**Affiliations:** 1 Department of Biotechnology, Alagappa University, Science Campus, Karaikudi, 630003 Tamil Nadu India; 2 School of Chemical Engineering, Yeungnam University, Gyeongsan, 38541 Republic of Korea; 3 ITC Life Sciences and Technology Centre, Bengaluru, India

**Keywords:** Biofilms, Infection

## Abstract

Methicillin resistant *Staphylococcus aureus* (MRSA) is a predominant human pathogen with high morbidity that is listed in the WHO high priority pathogen list. Being a primary cause of persistent human infections, biofilm forming ability of *S. aureus* plays a pivotal role in the development of antibiotic resistance. Hence, targeting biofilm is an alternative strategy to fight bacterial infections. The present study for the first time demonstrates the non-antibacterial biofilm inhibitory efficacy of 5-Dodecanolide (DD) against ATCC strain and clinical isolates of *S. aureus*. In addition, DD is able to inhibit adherence of MRSA on human plasma coated Titanium surface. Further, treatment with DD significantly reduced the eDNA synthesis, autoaggregation, staphyloxanthin biosynthesis and ring biofilm formation. Reduction in staphyloxanthin in turn increased the susceptibility of MRSA to healthy human blood and H_2_O_2_ exposure. Quantitative PCR analysis revealed the induced expression of *agrA* and *agrC* upon DD treatment. This resulted down regulation of genes involved in biofilm formation such as *fnbA* and *fnbB* and up regulation of *RNAIII*, *hld*, *psmα* and genes involved in biofilm matrix degradation such as *aur* and *nuc*. Inefficacy of DD on the biofilm formation of *agr* mutant further validated the *agr* mediated antibiofilm potential of DD. Notably, DD was efficient in reducing the *in vivo* colonization of MRSA in *Caenorhabditis elegans*. Results of gene expression studies and physiological assays unveiled the *agr* mediated antibiofilm efficacy of DD.

## Introduction

*Staphylococcus aureus* is an important human pathogen, which plays a remarkable role in an array of infections from negligible skin infections to life concerning invasive illness such as bacteremia, infective endocarditis etc^1^. Apart from the infections affiliated with vital organs/tissues, prosthetic device related infections are instigated predominantly by *S. aureus* infections. The ability to form biofilm on both biotic and abiotic surfaces is the key factor for the success of *S. aureus* in infections related to indwelling medical devices. Through a retrospective analysis, prevalence of *S. aureus* was observed in several implant associated infections^2^. Protein components of the bacterial cell wall facilitate the adhesion of *S. aureus* to the abiotic surfaces thereby establish persistent infections through biofilm formation. The secreted extracellular polymeric substances specifically extracellular DNA (eDNA), extracellular proteins, lipids, amyloid fibrils and polysaccharides such as polysaccharide intracellular adhesin (PIA) provide firm organization to the biofilm matrix. These biomolecules get absorbed on the implant surface providing initial attachment to the bacterial cell for the establishment of biofilm^3,4^. Staphylococcal cells encased within this biofilm matrix are extremely resistant to available antimicrobial therapy as well as to host immune responses^5^. Besides this, Quorum Sensing (QS) system coordinates the attachment and dispersal of biofilm cells by gene regulation^6^. This complex network enhances the ability of *S. aureus* to acquire resistance against wide spectrum of antibiotics including Methicillin, thereby making the conventional antibiotic treatment ineffective. Emergence of hospital and community associated MRSA has gained greater attention from public health sector and potentiate the need for novel therapeutic strategies^7^.

Methicillin resistance plays a crucial role in the phenotype of staphylococcal biofilms. In methicillin susceptible *S. aureus* (MSSA), biofilm formation is mediated by the production of *ica* operon encoded PIA. In contrast, *ica* independent biofilm formation has been described in MRSA and it is primarily mediated by the production of eDNA and cell surface adhesion proteins^8,9^.

Staphylococcal two component regulatory system (TCRS) encoded by accessory gene regulator (*agr*) acts as prototype QS system which controls the expression of major virulence genes according to the cell density in the surrounding environment^10^. In contrast to QS in other bacteria, active QS system in *S. aureus* inhibits the biofilm development by producing the matrix degrading enzymes. A previous study demonstrated that *agr* deficient *S. aureus* formed robust biofilm when compared to wild type. Altogether, *agr* mediated QS acts as a regulatory switch between planktonic and biofilm lifestyle of Staphylococcal cells^11^.

The search for novel therapeutic strategies such as use of antibiofilm agents or quorum sensing inhibitors provides a new insight in treating the recurrent and prolonged infections. In the past, coating antibiotics in combination with or without synthetic antibiofilm agents on implant surface in preoperative or postoperative conditions have been conducted to minimize the implant associated infections^12–14^. Extensive use of antibiotics can create a selective pressure in organisms and thereby resulting in development of antibiotic resistance. Thus, complementing antibiotics or synthetic antibiofilm agents with natural compounds will progress the therapy for persistent bacterial infections.

The present study is aimed at unveiling the antibiofilm efficacy of a food flavoring agent 5-Dodecanolide (DD) against a clinically important pathogen MRSA and to elucidate the mechanism underlying its antibiofilm activity.

## Results

### Screening of phytochemicals for antibiofilm activity against MRSA

**Figure 1:**
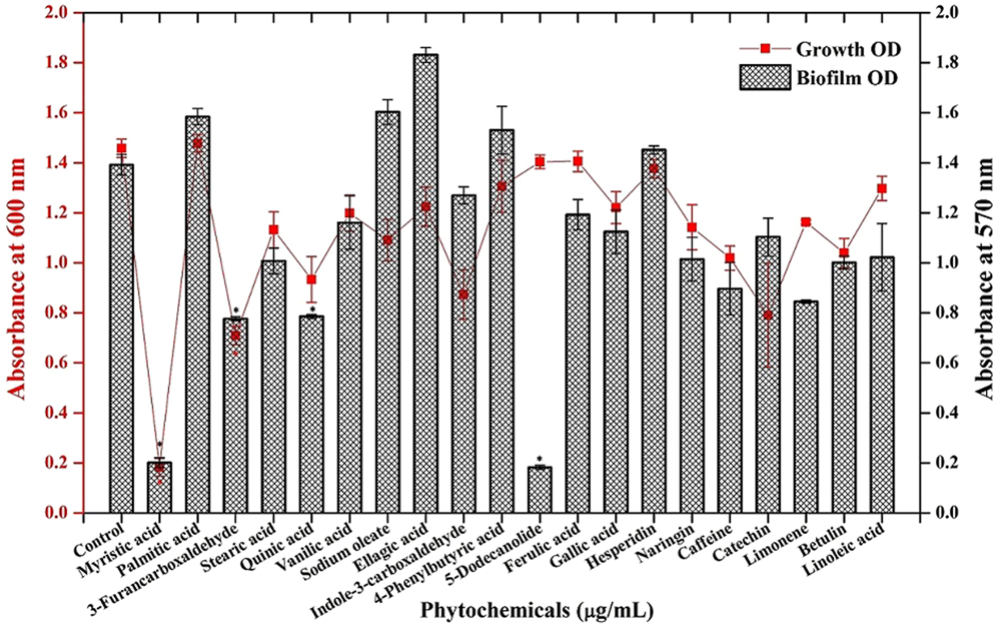
Screening of various phytochemicals for antibiofilm activity against MRSA. Error bars indicate standard deviations. Asterisks represent statistical significance (P < 0.05).

Biofilm inhibitory potential of 20 different phytochemicals (250 µg/mL) was assessed by 24 well MTP (micro titre plate) assay coupled with crystal violet staining. As observed from Fig. 1, OD at 570 nm was greatly reduced upon treatment with myristic acid and DD whereas rest of the compounds slightly affected the biofilm formation as well as growth of MRSA. Among these two compounds, myristic acid had antibacterial activity as observed from OD at 600 nm whereas DD exerted its antibiofilm potential without affecting the growth (Fig. 1).

### Determination of Biofilm inhibitory concentration (BIC) of DD

**Figure 2:**
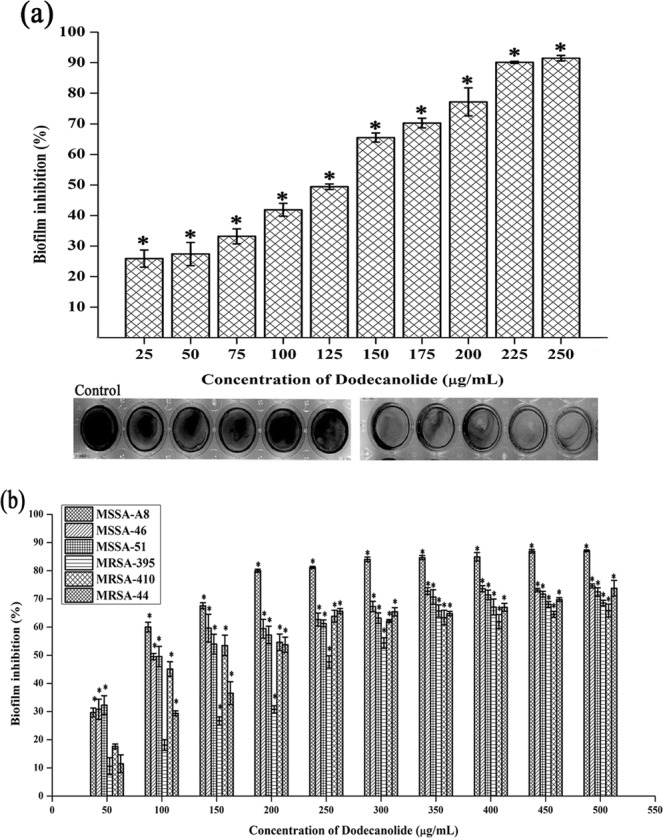
Determination of BIC of DD against MRSA reference strain (**a**) and clinical isolates (**b**) using crystal violet staining. Error bars indicate standard deviations. Asterisks represent statistical significance (P < 0.05).

For MRSA ATCC 33591, cells were treated with various concentrations of DD ranging from 25–250 µg/mL. Concentration dependent biofilm inhibition was observed upon DD treatment with maximum biofilm inhibition of 90% at 225 µg/mL concentration and hence this concentration was fixed as BIC of DD (Fig. 2a). For clinical isolates, DD treatment was administered in the range of 50–500 µg/mL. 200 µg/mL and 350 µg/mL were determined as BIC for MSSA A8 and MSSA 46, MSSA 51, MRSA 395, MRSA 410 and MRSA 44 respectively (Fig. 2b).

### Microscopic analysis of MRSA biofilm

**Figure 3:**
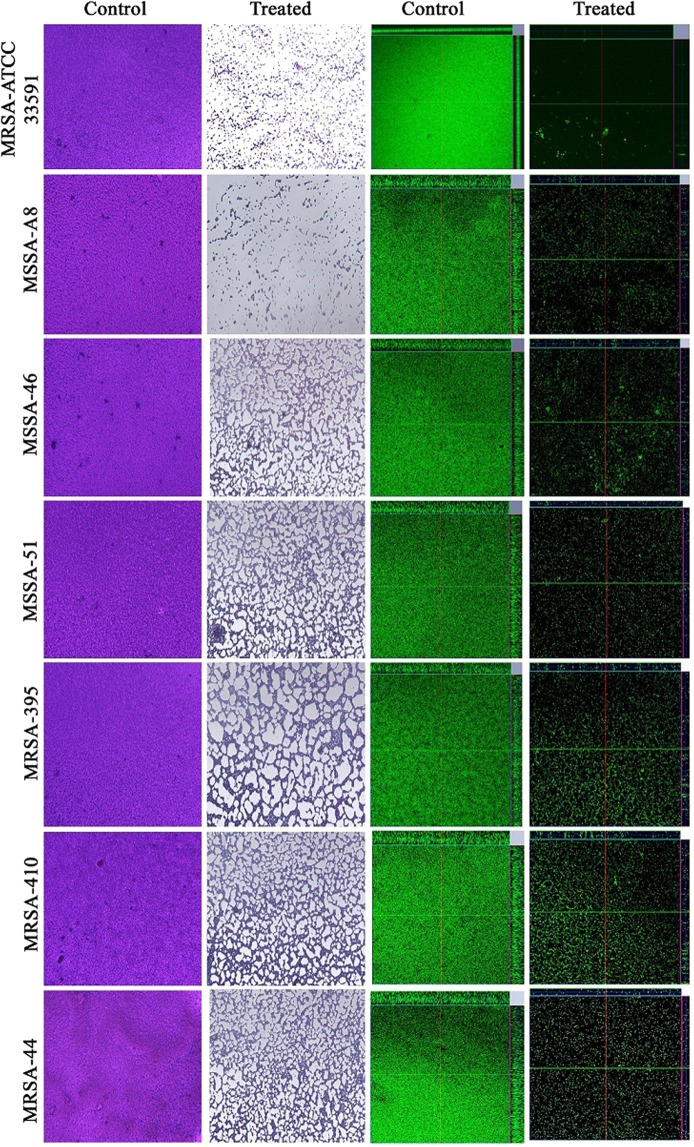
Light microscopic images (400X) and CLSM (200X) images depicting the antibiofilm potential of DD against tested *S. aureus* strains.

Antibiofilm potential of DD was further confirmed through light microscopic and confocal laser scanning microscopic (CLSM) analysis. Light microscopic images showed a great reduction in biofilm upon treatment with DD in all the strains tested. A completely covered dense biofilm was observed in case of control surface whereas disrupted biofilm with less number of cells was observed in the surface treated with BIC of DD. Further, architecture of biofilm was assessed by CLSM and the results confirmed the antibiofilm potential of DD. Upon treatment with BIC of DD, surface coverage, thickness of biofilm matrix and the number of cells adhered onto biofilm matrix were found to be greatly reduced compared to the control (Fig. 3).

###  Scanning electron microscope (SEM) analysis

**Figure 4:**
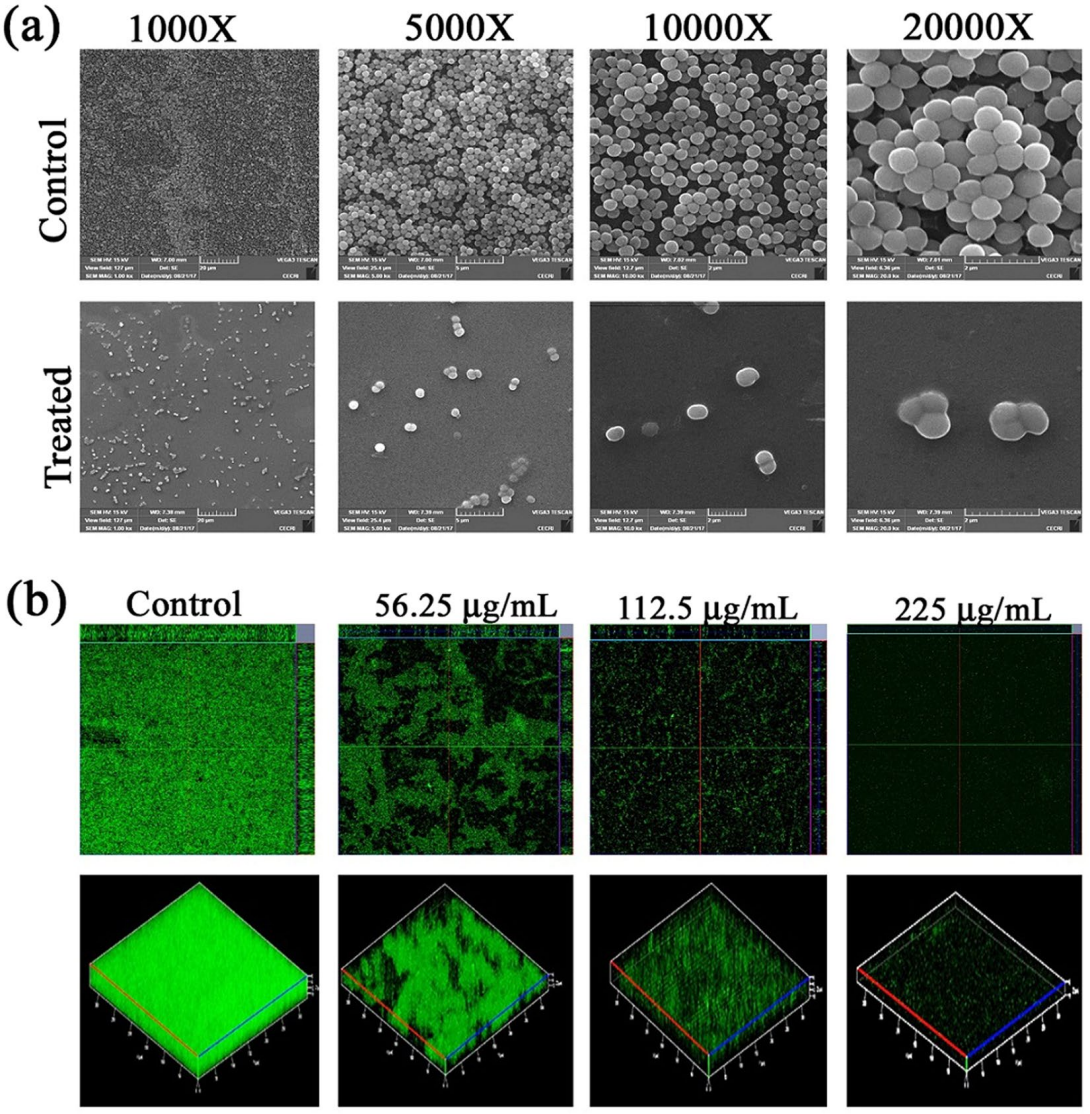
**(a)** SEM Micrographs of Control and DD treated MRSA biofilm showcasing the antibiofilm potential of DD on Ti surface. (**b**) Ortho and three-dimensional CLSM images revealing the reduction in MRSA biofilm formed on plasma coated Ti surface upon treatment with DD at increasing concentrations.

 SEM analysis was performed to elucidate the antibiofilm potential of DD on Titanium (Ti) surface. SEM images of control surface were completely covered with grape like clusters of staphylococcal cells. Multi-layer biofilm with microcolony formation was also observed. In case of DD treatment, biofilm formation was almost completely arrested and individually dispersed cells were observed (Fig. 4a).

### Effect of DD on MRSA biofilm formation on plasma coated Ti

The two and three dimensional CLSM micrographs of MRSA biofilms formed in the absence and presence of DD on plasma coated Ti slides showed biofilm inhibitory efficacy of DD on a concentration dependent manner (Fig. 4b).

### Effect of DD on growth and metabolism of MRSA

**Figure 5:**
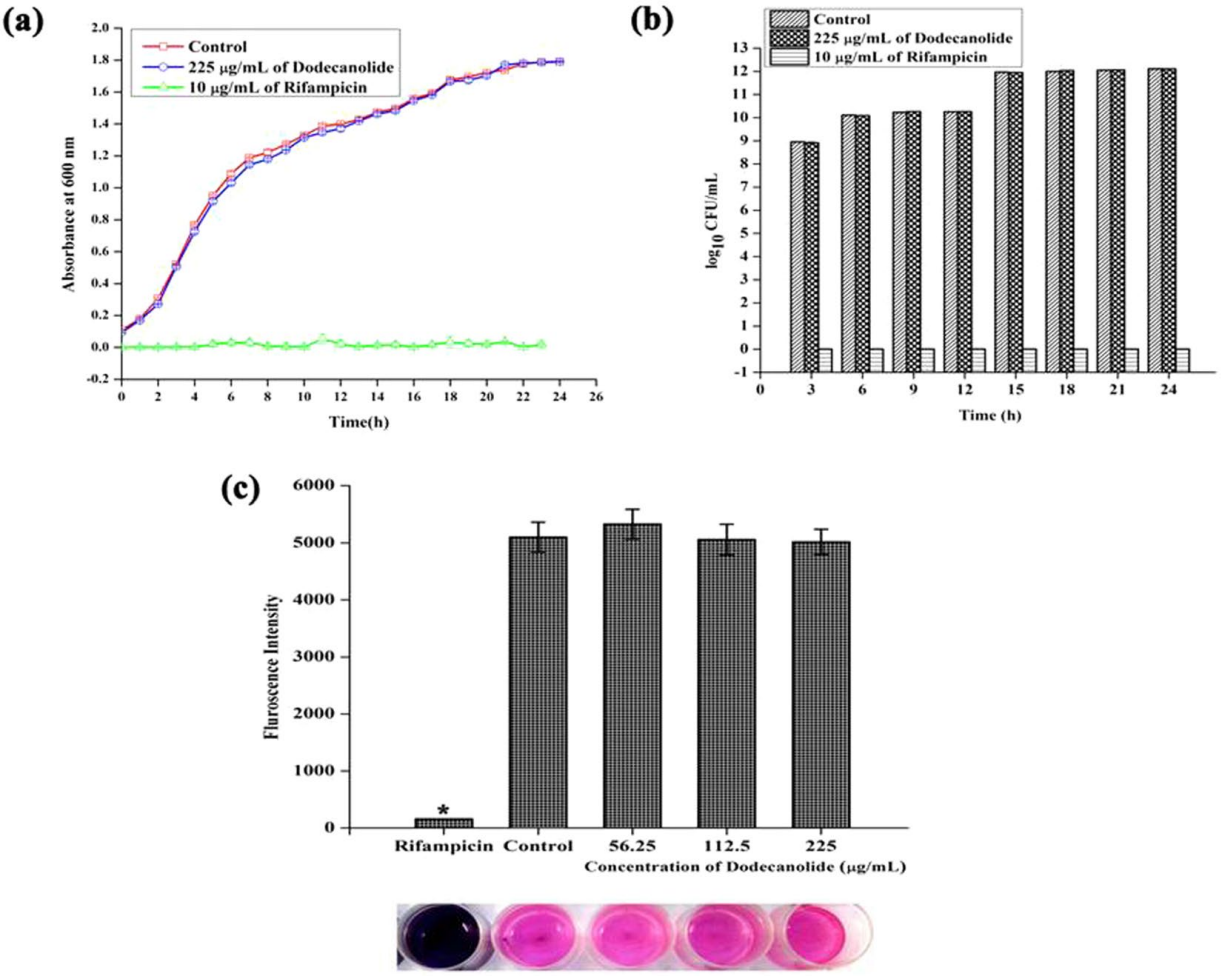
Effect of DD on growth and metabolism of MRSA at BIC (225 µg/mL). (**a**) Growth curve analysis. (**b**) CFU/mL of control, rifampicin and DD treated MRSA exhibiting non-antibacterial nature of DD at 225 µg/mL concentration. (**c**) DD treated MRSA cells were metabolically viable as comparable with control cells which was confirmed by alamar blue assay. Error bars indicate standard deviations.

In order to confirm the non-antibacterial nature of DD, growth curve analysis was performed in the absence and presence of DD and Rifampicin (positive control for growth inhibition). From the plotted growth curve, it is clear that DD do not interfere with the normal growth of the bacterium (Fig. 5a). CFU/mL data also confirmed the same. Therefore, it is neither bactericidal nor bacteriostatic in nature (Fig. 5b). Additionally, metabolic activity was measured using Alamar blue assay and it was found that DD treated cells are metabolically active as comparable to the control cells whereas Rifampicin treated cells are metabolically affected (Fig. 5c).

### Extracellular DNA (eDNA) extraction

**Figure 6:**
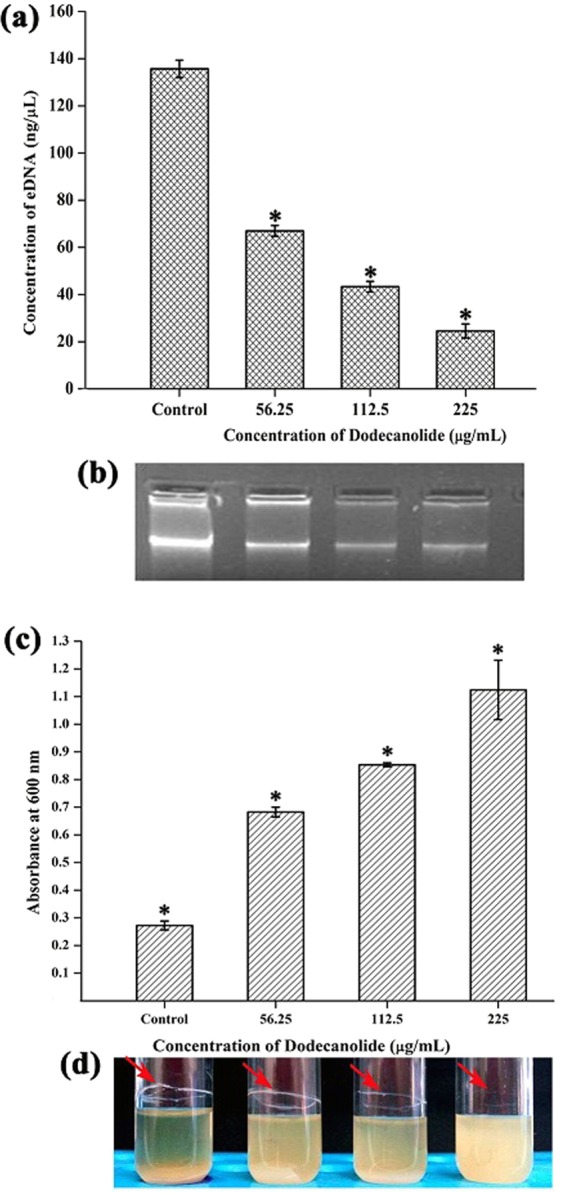
(**a**) DD treatment resulting in the reduction of eDNA present in MRSA biofilm in a concentration dependent manner. (**b**) Corresponding representation of agarose gel electrophoresis of eDNA. (**c**) Reduction in autoaggregation evidenced by increasing cell density at upper portion upon treatment with DD. (**d**) Corresponding representation of image of visual test tube settling assay. Red arrows indicate that dispersed cells are deficient in ring biofilm formation upon DD treatment when compared to aggregated control cells with ring biofilm. Error bars indicate standard deviations. Asterisks represent statistical significance (P < 0.05).

Concentration of eDNA present in control and DD treated samples given as bar graph indicates the reduction in eDNA (Fig. 6a). Agarose gel electrophoresis exhibited a concentration dependent reduction in eDNA synthesis upon DD treatment when compared with eDNA from control (Fig. 6b).

### Effect of DD on Autoaggregation of MRSA

Ability of MRSA to adhere and aggregate with the surrounding cells gives the stability to biofilm. Hence, the potential of DD to disrupt this intercellular aggregation was assessed using Visual test tube settling assay. The results showed that MRSA cells treated with DD were completely dispersed and also the ability of autoaggregation was observed to be reduced with increasing concentrations of DD (Fig. 6c,d).

### Effect of DD on staphyloxanthin synthesis, H_2_O_2_ sensitivity and whole blood survival of MRSA

**Figure 7:**
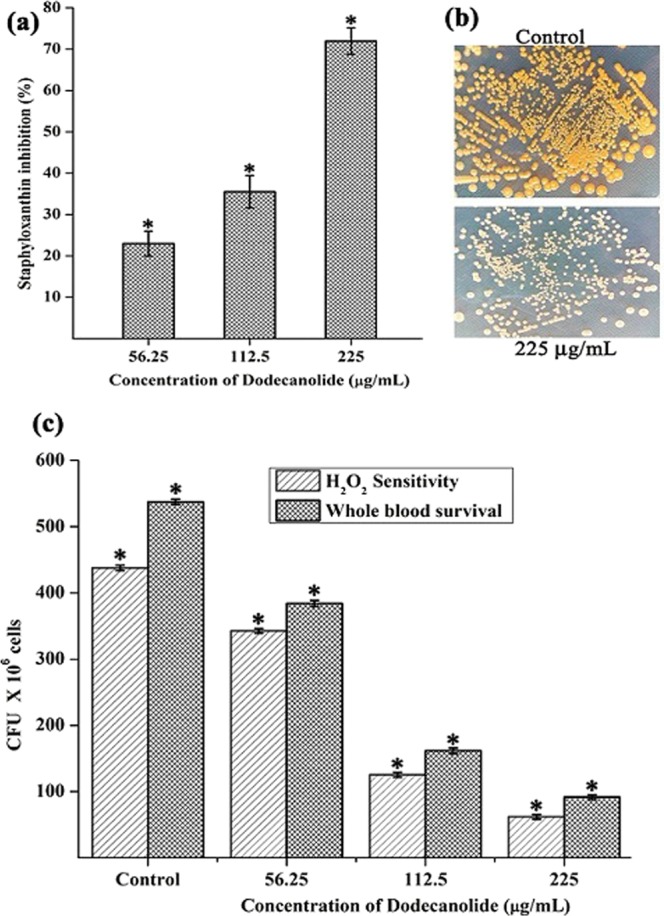
(**a**) Inhibition of staphyloxanthin pigment upon DD treatment in a dose dependent manner. (**b**) Images depicting the difference in production of staphyloxanthin pigment (golden yellow) by MRSA cells in the absence and presence of DD. (**c**) DD treatment reduced the survival of MRSA in the presence of H_2_O_2_ and in healthy human blood. Error bars indicate standard deviations. Asterisks represent statistical significance (P < 0.05).

Staphyloxanthin is the golden yellow colour pigment produced by MRSA and serves as one of the warriors of antioxidant defense system of MRSA. Effect of DD on pigment biosynthesis was assessed by methanol extraction protocol. Results unveiled the pigment inhibiting potential of DD to the extent of 71% (Fig. 7a,b). As DD impairs the antioxidant defense system of MRSA by inhibiting staphyloxanthin biosynthesis, it is expected to sensitize the MRSA cells to reactive oxygen species (ROS). Notably, generating ROS is the standard tactics of host neutrophils to kill the invading pathogens. Thus, H_2_O_2_ sensitivity assay and whole blood survival analysis were performed to check the ability of DD to sensitize MRSA cells towards ROS. As expected, DD greatly reduced the number of cells resistant to H_2_O_2_ as well as the number of cells survived in whole blood when compared to untreated control cells (Fig. 7c).

### Gene expression analysis by real time PCR

**Figure 8:**
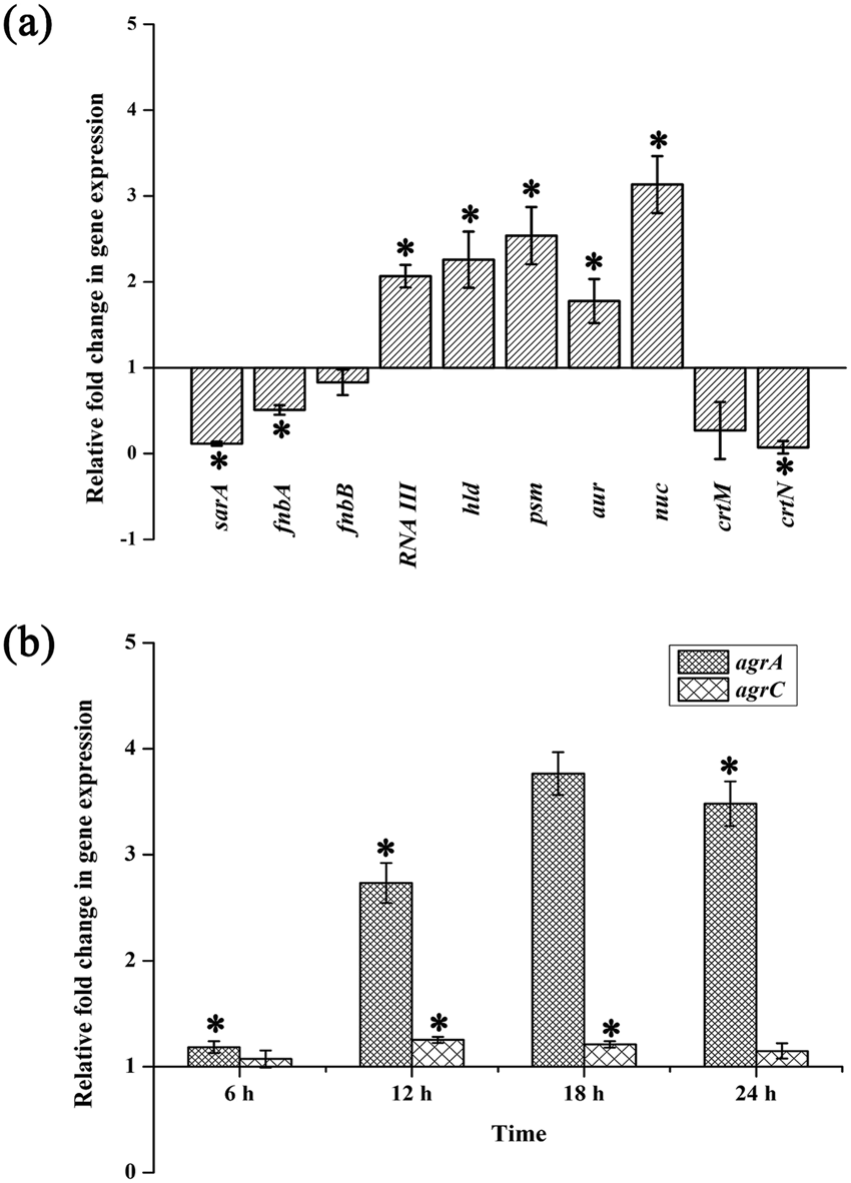
(**a**) Expression of genes involved in biofilm and virulence after 24 h DD treatment. (**b**) Expression of *agrA* and *agrC* upon DD treatment at 6 h time interval. Error bars indicate standard deviations. Asterisks represent statistical significance (P < 0.05).

In order to analyze the effect of DD at molecular level, gene expression analysis of important regulatory genes and virulence genes was performed using real time PCR. The gene expression profile of treated cells was compared with that of control cells and the fold change was calculated. Expression profile of master regulator genes *agrA* and *agrC* was assessed at 6 h, 12 h 18 h & 24 h and found to be up regulated at all the tested time points (Fig. 8b). Expression of *agr* regulated and other virulence genes was analyzed only after 24 h. Up regulation was found in the expression levels of *RNAIII*, *hld*, *psmα*, *aur* and *nuc* whereas down regulation was observed in case of *sarA*, *fnbA*, *fnbB*, *crtM* and *crtN* (Fig. 8a).

### DNase assay

**Figure 9:**
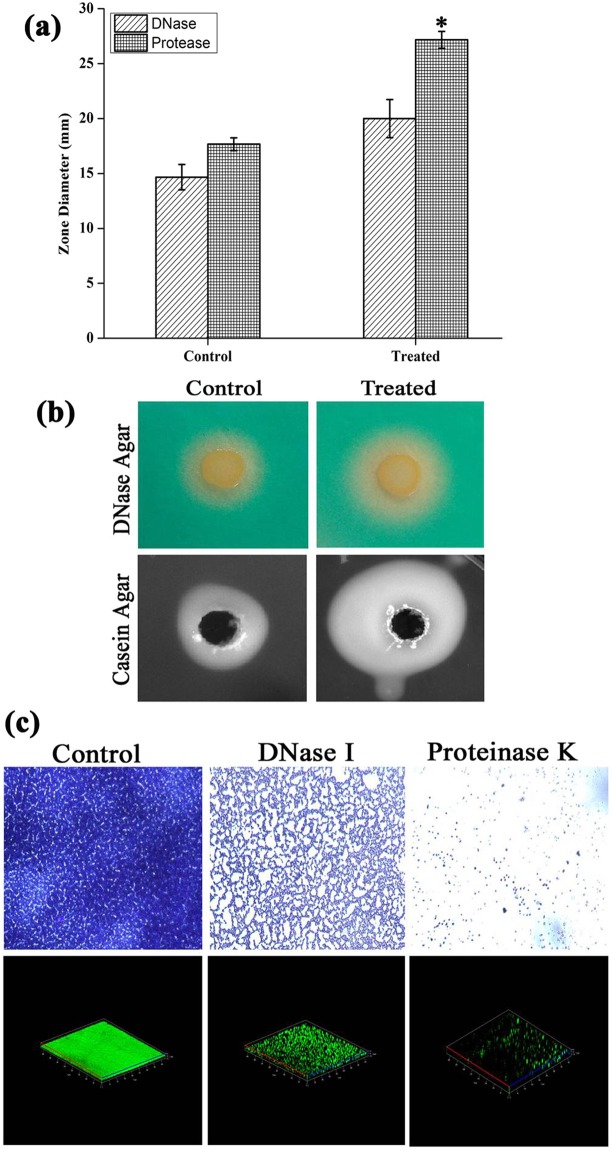
(**a**) Assessment of extracellular protease and DNase activity revealing the increased zone diameter upon DD treatment. (**b**) Representative images of enzymatic activity in the absence and presence of DD. (**c**) Light microscopic and CLSM images of MRSA biofilm in the absence and presence of exogenous enzymes proteinase K and DNase I unveiling the significance of eDNA and eProteins. Error bars indicate standard deviations. Asterisks represent statistical significance (P < 0.05).

In DNase agar plates, zone of clearance around cells against green background was observed as a result of DNA hydrolysis. Interestingly, the zone diameter is relatively higher in DD treated cells (20 mm) than control cells (14 mm). Hence, from the result it is clear that DD treatment increased the DNase production which is in line with the results of eDNA extraction assay where eDNA was greatly reduced in treated sample (Fig. 9a,b).

### Protease assay

In Protease agar plates, as an indication of extracellular protease activity, the white zone was observed around the well after incubation period. The diameter of white zone was observed to be 18 mm and 27 mm in control and treated plates respectively. This result clearly depicts the increase in extracellular protease production upon DD treatment (Fig. 9a,b).

### Exogenous enzymatic treatment

Light microscopic and CLSM images clearly depicted the reduction in biofilm upon treatment with exogenous enzymes. Notably, proteinase K treatment completely inhibited the formation of MRSA biofilm thereby revealed the importance of proteins in biofilm matrix. DNase I treatment also considerably reduced the biofilm which implies that eDNA also plays a critical role in biofilm matrix (Fig. 9c).

### Effect of DD on biofilm formation by *agr* mutant

**Figure 10:**
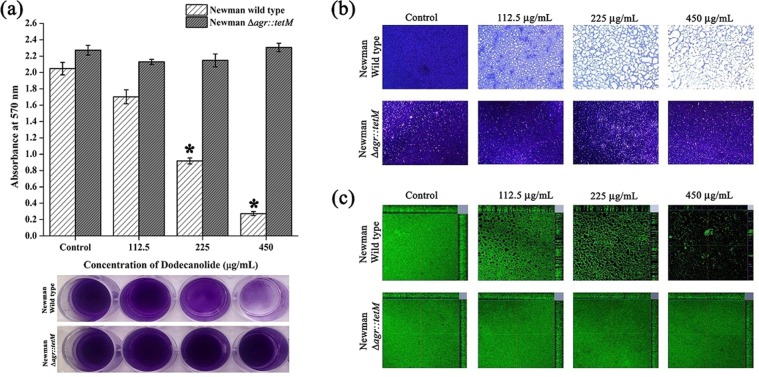
Effect of DD on biofilm formation of Newman wild type strain and isogenic *agr* mutant assessed by (**a**) crystal violet quantification, (**b**) light microscopic and (**c**) CLSM analysis. Error bars indicate standard deviations. Asterisks represent statistical significance (P < 0.05).

The results of crystal violet quantification, light microscopic and CLSM showed inefficacy of DD against biofilm formation by *agr* mutant strain ALC 355 whereas DD was found to be effective against isogenic wild type strain at 450 µg/mL concentration (Fig. 10).

### Effect of DD on *in vivo* Biofilm of MRSA

**Figure 11:**
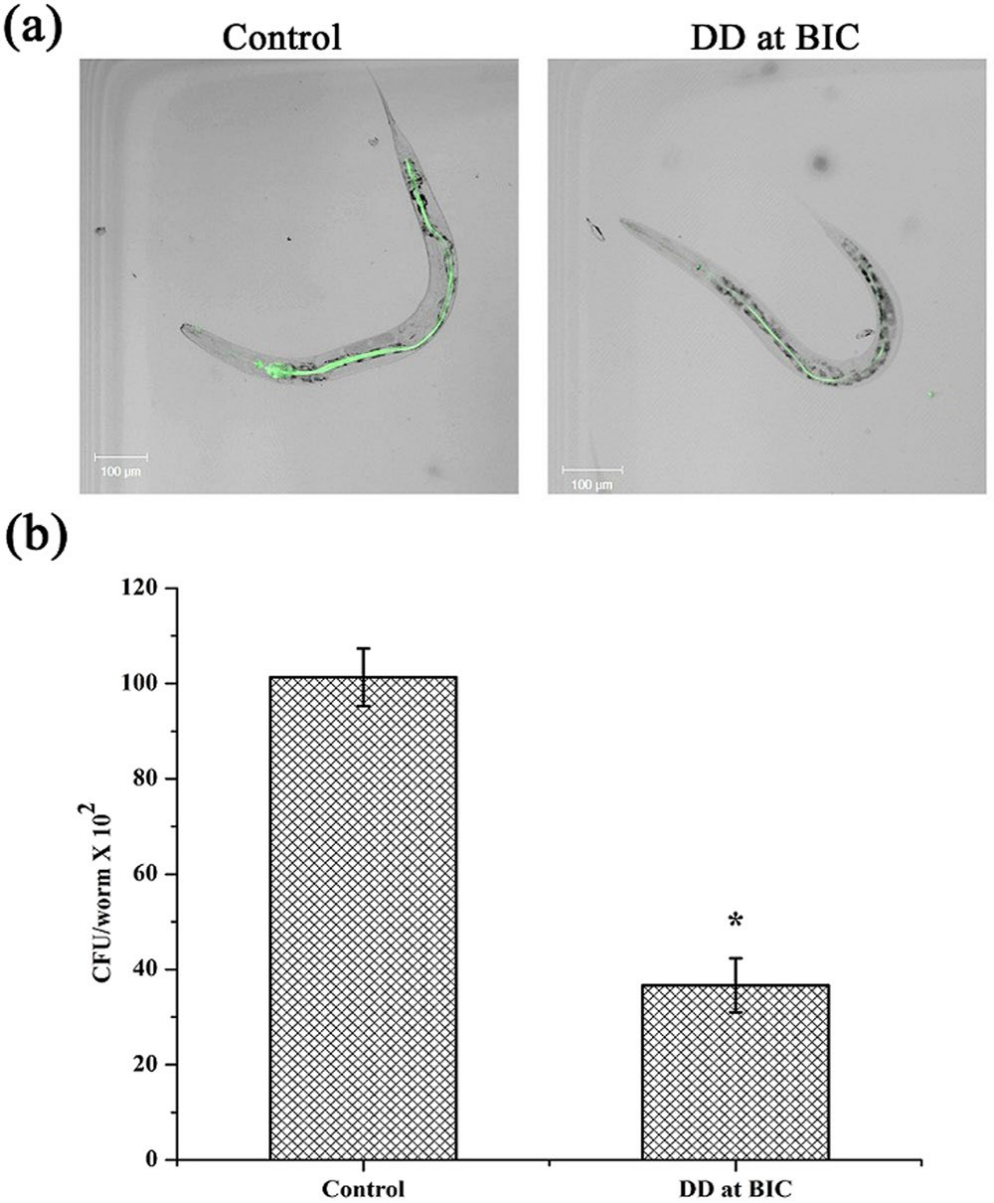
Effect of DD on *in vivo* biofilm formation (**a**) CLSM micrograph showing internal colonization of MRSA in *C. elegans*. Amount of fluorescence is directly proportional to the amount of bacterial colonization (**b**) CFU analysis representing the reduced bacterial count upon DD treatment. Error bars indicate standard deviations. Asterisks represent statistical significance (P < 0.05).

*In vivo* antibiofilm potential of DD was assessed using the *C. elegans* – MRSA infection model. On CLSM analysis, it was observed that the nematodes challenged with the test pathogen displayed visible MRSA Biofilm in the intestinal region whereas the nematodes challenged with MRSA and treated with DD showed reduction in the colonization of bacterial cells in the intestinal region (Fig. 11a). In CFU assay, DD treatment reduced the colonized bacterial load to 36.67 × 10^2^ cells when compared with the bacterial load 101.33 × 10^2^ cells in control nematodes. This result clearly demonstrated approximately 64% reduction in the internal colonization of MRSA upon treatment with DD (Fig. 11b).

## Discussion

*S. aureus* is a human commensal bacterium, which turns into a notorious pathogen when gets supportive opportunity^1^. Once turned into opportunistic pathogen, it produces an array of colonizing proteins and virulence determinants to establish infection by forming robust biofilm^15^. Biofilm formation is reported to be the major cause for the failure of antibiotic treatment^9^. Hence, inhibition of biofilm formation will prevent the progression of infection and also sensitize the bacterial cells towards host immune clearance and antibacterial therapy as well. In the current era, finding the antibiofilm agents has become an alternative strategy to fight bacterial infections without exerting any negative impact on growth and metabolic viability of bacteria^16^. In the present study, 20 different phytochemicals were screened at 250 µg/mL concentration for antibiofilm activity and 5-Dodecanolide was found to be the potential compound in inhibiting biofilm formation without affecting growth of MRSA (Fig. 1). 5-Dodecanolide (Pubchem CID: 12844) is a lactone group of compound present in few fruits in low abundance and a well known food flavoring agent used in most of the dairy products for aroma^17,18^. Due to its less natural abundance, it is chemically synthesized and utilized in food industry. For the first time, the present study explored the antibiofilm potential of DD against *S. aureus*. Crystal violet quantification of biofilm formed on polystyrene surface exhibited the dose dependent antibiofilm activity of DD. 225 µg/mL, 200 µg/mL and 350 µg/mL were the concentrations which exhibited maximum biofilm inhibition against MRSA ATCC strain, MSSA-A8 and other clinical isolates MSSA 46, MSSA 51, MRSA 395, MRSA 410 and MRSA 44 respectively and the same concentrations were determined as BIC of DD (Fig. 2a,b). Antibiofilm efficacy of BIC of DD on glass surface was tested by light microscopy and CLSM analysis. Both analyses showed a great reduction in biofilm covered surface area and thickness in all tested strains of *S. aureus* (Fig. 3). Hence, the broadness of biofilm inhibitory potential of DD was witnessed through biofilm assays against various strains of *S. aureus* and MRSA ATCC reference strain was taken for further analysis. *S. aureus* has been reported to be a predominant pathogen associated with indwelling medical device associated infections^19^. As colonization of MRSA on metal surface is a serious clinical issue, efficacy of DD in inhibiting biofilm formation on Ti surface was evaluated by SEM analysis. Results exhibited the great reduction in MRSA colonization on Ti surface in the presence of DD (Fig. 4a). Previous studies reported that implanted medical devices were coated with host plasma proteins which made the metal surface more suitable for adherence and accumulation of bacterial cells^20,21^. Thus, biofilm inhibitory potential of DD on human plasma coated Ti surface was examined by CLSM analysis. Remarkably, DD was efficient in inhibiting biofilm formation even on plasma coated Ti surface in a dose dependent manner (Fig. 4b). Thus, inhibition of biofilm formation on all the tested surfaces irrespective of their surface chemistry makes DD as potential antibiofilm agent in clinical setting as most of the nosocomial infections arise out of the biofilm formed on medical devices and implants. Formation of biofilm is an intricate phenomenon involving various stages namely initial attachment, micro colony formation, maturation and dispersal. The surface independent antibiofilm potential of DD suggests that DD probably interferes with initial adhesion. Non-antibacterial nature of DD at BIC was confirmed by growth curve analysis (both OD and CFU/mL data) and Alamar blue assay for control and treated MRSA cells (Fig. 5a–c). No significant reduction in growth was observed in growth curve analysis which was further substantiated by metabolically viable treated cells as compared with control cells in Alamar blue assay whereas rifampicin completely arrested the growth and viability of the MRSA. Therefore, it is to be construed that DD exhibits non antibacterial antibiofilm activity which is an important criterion to exclude the resistance development. Biofilm is the multi-component matrix majorly comprising of exopolysaccharides (EPS), extracellular proteins (eProteins) and eDNA^22^. Controlled lysis of sub population of *S. aureus* biofilm releases their genomic DNA into extracellular environment, and it is named as eDNA^23^. eDNA is reported to play a crucial role in biofilm formation by providing the structural integrity to the biofilm matrix^24^. Therefore, DNA extracted from control and treated biofilm matrix was quantified and visualized after agarose gel electrophoresis. DD treatment greatly reduced the eDNA quantity in a dose dependent manner (Fig. 6a,b). eDNA mediated intracellular adhesion was reported as an important mechanism behind autoaggregation in MRSA^25^. This fact driven the study to assess the autoaggregation pattern of MRSA in the presence of DD. Results of visual test tube settling assay unveiled the complete dispersal of DD treated cells (Fig. 6c,d). Notably, dispersed cells are deficient in ring biofilm formation when compared to autoaggregated cells with ring biofilm (control). Hence, reduction in eDNA impacted the biofilm formation by interrupting the intracellular adhesion. In all the assays performed, DD treated cells appeared white in color when compared to yellow colored control cells (Fig. 7a,b). Appearance of yellow color is due to the production of carotenoid pigment Staphyloxanthin. Staphyloxanthin located in the cell membrane provides physical fitness to MRSA cells by scavenging ROS^26^. Being a warrior of antioxidant defensive system, Staphyloxanthin helps MRSA to escape from oxidative stress generated by human neutrophil of the host immune system^27,28^. Thus, the inhibition of Staphyloxanthin biosynthesis prompted to study the effect of DD on survival of MRSA in whole human blood and H_2_O_2_ sensitivity. As anticipated, DD treatment profoundly reduced the number of cells survived in the healthy human blood and H_2_O_2_ treatment (Fig. 7c). It can be affirmed that DD treatment sensitize the MRSA cells to human netutrophil mediated killing by inhibiting the biosynthesis of staphyloxanthin.

In order to identify the molecular mechanism of DD at transcriptome level, real time PCR was performed for major biofilm, virulence and master regulator genes (Fig. 8a). Effect of DD on enzymes involved in the synthesis of Staphyloxanthin, 4,4′-diapophytoene synthase and 4,4′-diapophytoene desaturase encoded by *crtM* and *crtN* respectively was examined and it was found that DD effectively down regulated the expression of *crtM* and *crtN* which could be responsible for reduction in Staphyloxanthin^29^.

Impact of DD on one of the master regulators namely staphylococcal accessory regulator A (*sarA*) was also investigated as *sarA* locus has been reported to play a critical role in regulation of virulence genes of *S. aureus*^30^. DD treatment down regulated the expression of *sarA* and this can also be possible mechanism behind the antibiofilm activity of DD as previous studies reported the antibiofilm activity of Sar A inhibitors against *S. aureus*^31^.

MRSA produces numerous adhesion proteins to colonize the target surface and these adhesive proteins are termed as the microbial surface component recognizing adhesive matrix molecules (MSCRAMM)^32^. Among the identified MSCRAMM molecules, fibronectin binding proteins (FnBPs) are well characterized and primarily involved in tissue colonization^33^. Hence, expression of primary adhesion proteins FnbA and FnbB was studied and found to be down regulated upon DD treatment. Based on the obtained data, it is hypothesized that DD inhibits the biofilm formation at the initial stage by reducing the expression of adhesion proteins. In *S. aureus*, QS circuit is well connected by the presence of TCRS comprising of receptor kinase AgrC and response regulator AgrA^5^. It has already been reported that active QS system hampers the initial attachment and biofilm development and *agr* mutant strains could form vigorous biofilms compared to wild type strains^11^. *agr* system impedes the biofilm development by suppressing the production of adhesion proteins and inducing the expression of matrix degrading enzymes such as protease, nuclease and lipase^11^. In the present study, DD up regulated the expression of *agrA* and *agrC*, thereby activates QS circuit which resulted in the down regulation of adhesion proteins FnbA and FnbB, whereas induced the expression of aureolysin and nuclease respectively. These results are in line with the previous study, where *agr* mediated dispersal of *S. aureus* biofilm by secretion of extracellular proteases has been demonstrated^7,34^. In addition, expression of *agr* controlled virulence genes namely *RNAIII*, *hld* and *psmα* was also found to be up regulated^35^.

In order to confirm the phenotypic expression of secreted enzymes, protease and DNase assay were performed. Results revealed that DD induced the expression of protease as well as nuclease when compared to control (Fig. 9a,b). In order to prove the fact that secreted enzymes play a pivotal role in antibiofilm efficacy of DD, biofilm architecture was analyzed using light microscope and CLSM analysis upon addition of exogenous proteinase K and DNase I to the medium. Biofilm inhibition by proteinase K treatment was observed to be efficacious when compared to the biofilm inhibition by DNase I (Fig. 9c). This result is in agreement with the earlier work of biofilm inhibition by exogenous enzymes^36^. Though *sarA* is also down regulated upon treatment with DD, consistent *agr* up regulation as observed from qPCR analysis at different time points (Fig. 8b) and phenotypic assays suggest that the *agr* mediated antibiofilm activity to be the possible mechanism of DD. As increased expression of *agr* inhibits biofilm formation, absence of *agr* should enhance biofilm formation. In a previous study, *agr* mutants were reported to be robust biofilm formers^37^. Hence, If *agr* up regulation is the mechanism behind antibiofilm efficacy of DD, then *agr* mutants should be resistant to DD. In order to ascertain this fact, antibiofilm efficacy of DD on Newman wild type strain and isogenic *agr* mutant was examined (Fig. 10). Interestingly, DD was found to be effective against biofilm formation of wild type strain whereas it was ineffective against biofilm formation of *agr* mutant. Crystal violet quantification revealed a dose dependent antibiofilm activity of DD against wild type strain with maximum biofilm inhibition at 450 µg/mL whereas *agr* mutant strain was resistant to DD treatment at all tested concentrations (Fig. 10a). Light and CLSM micrographs (Fig. 10b,c) further validated the results of crystal violet quantification. As DD was unable to exert its antibiofilm activity in *agr* mutant, it can be concluded that up regulation of *agr* could be the primary mechanism behind the antibiofilm activity of DD.

*C. elegans* has been extensively accepted as an animal model to evaluate host–pathogen interactions such as pathogenesis, immune defense and *in vivo* biofilm infections and it shares numerous cellular and molecular pathways with human^38,39^. More specifically, *C. elegans* has been used to study the pathogenesis and *in vivo* biofilm formation of *S. aureus* and *S. epidermidis*^40,41^. Hence, potential of DD as an effective antibiofilm agent under *in vivo* conditions was assessed by *C. elegans* infected with MRSA. The CLSM micrographs of infected *C. elegans* in the absence and presence of BIC of DD clearly displayed decreased biofilm formation of the bacterial cells in the intestinal region in DD treated nematodes (Fig. 11a). CFU assay further substantiated the *in vivo* antibiofilm potential of DD observed through the microscopic visualization (Fig. 11b). From these results, it is apparent that DD effectively reduces the adherence of MRSA cells to the intestine of the nematode, which strongly evidences the *in vivo* antibiofilm potential of DD.

## Materials and Methods

### Ethical statement

In the present study, healthy human blood was used for whole blood survival assay and plasma coating. The blood sample from healthy human (one of the authors of the manuscript) was drawn by a technically trained person and a written informed consent was obtained. The experimental protocol and the use of healthy human blood was assessed and approved by the Institutional Ethical Committee, Alagappa University, Karaikudi (IEC Ref No: IEC/AU/2016/1/4). All methods were carried out in accordance with relevant guidelines and regulations.

### Bacterial strain and growth conditions

*S. aureus* strains used in this study (as given in Table 1) were cultured at 37 °C on Tryptone soya agar (TSA) and maintained at 4 °C. For biofilm and virulence assays, Tryptone soya broth supplemented with 1% sucrose (TSBS) was used. Clinical isolates of *S. aureus* were collected from pharingitis patients^42^.

**Table 1:** *S. aureus* strains used in the present study.

Strain Name	Details
MRSA ATCC 33591	Reference strain obtained from ATCC
Newman Wild type	Reference strain NCTC 8178
ALC 355	Newman ∆*agr*::*tetM*
MSSA A8	GenBank ID: JN315152
MSSA 46	GenBank ID: JN315153
MSSA 51	GenBank ID: JN315154
MRSA 395	GenBank ID: JN390832
MRSA 410	GenBank ID: JN315150
MRSA 44	GenBank ID: JN315148

### Compounds used

Myristic acid, Palmitic acid, 3-Furan carboxaldehyde, Stearic acid, Quinic acid, Vanilic acid, Sodium oleate, Indole -3-carboxaldehyde, 4-phenyl butyric acid, 5-Dodecanolide (DD), Ferulic acid, Gallic acid, Naringin, Caffeine, Catechin, Limonene, and Linoleic acid were dissolved in methanol (10 mg/mL). Hesperidin, Ellagic acid and Betulin were dissolved in DMSO (10 mg/mL). Methanol was used as vehicle control in all the assays were DD was used.

### Screening of phytochemicals for antibiofilm activity against MRSA

Totally 20 phytochemicals were screened for their antibiofilm activity using 24 well MTP assay. Each well was loaded with 1 mL of TSBS with 1% overnight grown bacterial culture (Initial Optical Density (OD) 0.1 at 600 nm). For screening, 250 µg/mL of each compound was added and assay plate was kept at 37 °C for 24 h. After incubation period, OD at 600 nm was measured. Then planktonic cells were discarded and each well was washed with sterile water to remove loosely attached cells and then the plate was air dried. For quantification, biofilm cells in each well was stained with 0.4% of crystal violet for 10 min and excess stain was removed by washing plates twice with distilled water and air dried. Biofilm cells were destained using 10% glacial acetic acid and OD was read at 570 nm using multi-label reader (Spectramax M3, USA)^43^.

### Determination of BIC of DD

To determine the BIC of DD, 24 well MTP assay was performed as mentioned earlier with various concentrations of DD ranging from 25–250 µg/mL for MRSA ATCC 33591 and 50–500 µg/mL for clinical isolates. Well containing TSBS + MRSA+ methanol was maintained as control. The absorbance of control and treated wells was measured at 570 nm. The percentage of biofilm inhibition was calculated using the formula:

$$ \% \,{\rm{of}}\,{\rm{inhibition}}=[({\rm{Control}}\,{{\rm{OD}}}_{570{\rm{nm}}}-{\rm{Treated}}\,{{\rm{OD}}}_{570{\rm{nm}}})/{\rm{Control}}\,{{\rm{OD}}}_{570{\rm{nm}}}]\times 100.$$

### Microscopic analysis of biofilm

For microscopic analysis, bacterial cells were allowed to form biofilm on glass slides/Ti surface (1 cm × 1 cm) in the absence and presence of DD for 24 h at 37 °C and then washed with sterile distilled water and stained as needed.

For light microscopic analysis, the washed glass slides were stained with 0.4% crystal violet and washed to remove the excess stain and then air dried. Finally, the glass slides were examined at magnification of 400X under light microscope (Nikon Eclipse 80i, USA).

For CLSM analysis, the washed glass slides were stained with 0.1% of Acridine orange for 10 min at dark followed by washing and drying which was then observed under CLSM (LSM 710, Carl Zeiss, Germany) at magnification of 200X.

For SEM analysis, the washed Ti plates were fixed with glutaraldehyde solution (2%) at 4 °C for 8 h. After incubation period, the Ti plates were washed. Further, the Ti plates were dehydrated using increasing concentrations of ethanol (20, 40, 60, 80 and 100%) and allowed to air dry. Gold sputtering was done at vacuum condition prior to observation under SEM (VEGA 3 TESCAN, Czech Republic)^44^.

### Preparation of plasma coating on Ti

Plasma collected from human blood sample was diluted to a final concentration of 20% in 50 mM sodium bicarbonate. 1 cm × 1 cm Ti slides were placed in 24 well MTP and covered with 1 mL of 20% plasma solution and incubated at 4 °C overnight. The next day, 20% plasma solution was removed and Ti slides were washed with sterile distilled water. Biofilm inhibitory potential of DD on plasma coated surface was assessed by MTP assay and observed under CLSM as mentioned earlier^45^.

### Growth curve analysis

To analyze the effect of DD on growth of MRSA, 100 mL of TSBS was inoculated with 1% overnight culture of MRSA in the absence and presence of BIC of DD and 10 µg/mL of rifampicin. Initial OD (0 h) was measured at 600 nm and the cultures were kept at 37 °C. OD values were taken at 1 h interval for 24 h and CFU/mL was calculated at 3 h interval for 24 h and the growth curve was plotted as OD against time interval along with CFU/mL^46^.

### Alamar blue assay

To analyze the effect of DD on cell viability and proliferation, alamar blue assay was performed. Briefly, control, DD (56.25 µg/mL, 112.5 µg/mL and 225 µg/mL) and rifampicin (10 µg/mL) treated cells were collected as pellet after centrifugation at 12,000 rpm for 15 min. Then the cell pellets were resuspended in 0.9% saline and mixed with 1/10 volume of Alamar blue (6 mg/mL) and incubated at 37 °C for 12 h. Finally, fluorescence intensity was measured at excitation and the emission wavelength of 530 nm and 590 nm respectively (Spectramax M3, USA)^47^.

### eDNA extraction

In 6 well MTP, 5 mL of TSBS was inoculated with 1% of MRSA in the absence and presence of DD (56.25 µg/mL, 112.5 µg/mL and 225 µg/mL). After 24 h of incubation, planktonic cells were carefully removed without disrupting the biofilm and 1 mL of TE buffer [pH 8] containing 10 mM Tris and 1 mM EDTA was added to biofilm. The biofilm matrix along with cells was completely scrapped out and transferred to 1.5 mL tubes which were then centrifuged at 12, 000 rpm for 10 min to remove supernatant. The settled biofilm pellet was resuspended in 200 µL of TE buffer and vortexed for 1 h to disengage the biofilm components. Again, the tubes were centrifuged to collect supernatant containing released eDNA. Finally, 20 µL of supernatant was run in 1% agarose gel and eDNA was visualized using ethidium bromide staining^25^. Also, the extracted eDNA was quantified using Bionano Spectrophotometer (Shimadzu, Kyoto, Japan).

### Autoaggregation assay

Autoaggregation was analyzed using visual test tube settling method as reported earlier. MRSA cultures were grown for 24 h in the absence and presence of DD (56.25 µg/mL, 112.5 µg/mL and 225 µg/mL) in the test tubes. After incubation period, the test tubes were taken out of shaker and kept statically for 30 min. The images were captured after incubation period^25^. Cell density of uppermost portion was assessed by measuring OD at 600 nm. Autoaggregation can be seen as settling of cells at the bottom of the tube.

### Staphyloxanthin assay

24 h cultures of MRSA grown in the absence and presence of DD (56.25 µg/mL, 112.5 µg/mL and 225 µg/mL) were centrifuged at 12,000 rpm for 5 min and supernatants were discarded. Cell pellets were subjected to methanol extraction and kept at 37 °C under shaking condition overnight. Extracted Staphyloxanthin was quantified by measuring OD at 462 nm^29^.

### Whole blood survival assay

MRSA cells grown in the absence and presence of DD (56.25 µg/mL, 112.5 µg/mL and 225 µg/mL) were added to healthy human blood in the ratio of 1:4. The mixture was incubated at 37 °C for 3 h with agitation. The viable cells were enumerated using spread plate method^27^.

### H_2_O_2_ killing assay

Bacterial cells were prepared as stated earlier. Collected cell pellets were suspended in 1 mL of phosphate buffered saline (PBS), subsequently treated with 0.2% of H_2_O_2_ and incubated at 37 °C for 3 h and then plated on TSA to analyze the viable bacterial cells. After overnight incubation, colonies formed were counted to plot the graph^48^.

### RNA extraction and cDNA synthesis

Total RNA was extracted from the control and DD (225 µg/mL) treated MRSA cells using Trizol method of extraction at 6 h interval for 24 h. And then RNA extracted from various time points was converted to cDNA using High Capacity cDNA Reverse Transcription Kit (Applied Biosystems, USA)^49^.

### Real time PCR analysis

Expression analysis was performed using real time PCR (7500 Sequence Detection System, Applied Biosystems Inc. Foster, CA, USA) for the candidate genes *agrA, agrC, sarA, fnbA, fnbB, aur, nuc, RNAIII, hld, psmα, crtM* and *crtN* involved in virulence and biofilm formation. PCR reaction was performed at predefined ratio using PCR Master Mix (SYBR Green kit, Applied Biosystems, USA). Expression pattern of candidate genes was obtained by calculating 2^−∆∆Ct^ after normalizing the Ct (cycle threshold) values of respective genes against the Ct value of the housekeeping gene (*gyrB*)^49^. Primer sequences of candidate genes are given in Table 2.

**Table 2:** List of primers used for Q-PCR analysis.

Genes	Forward primer	Reverse primer
*agrA*	5′-TGATAATCCTTATGAGGTGCTT-3′	5′-CACTGTGACTCGTAACGAAAA-3′
*agrC*	5′-CATTCGCGTTGCATTTATTG-3′	5′-CCTAAACCACGACCTTCACC-3′
*sarA*	5′-CAAACAACCACAAGTTGTTAAAGC-3′	5′-TGTTTGCTTCAGTGATTCGTTT-3′
*fnbA*	5′-ATCAGCAGATGTAGCGGAAG-3′	5′-TTTAGTACCGCTCGTTGTCC-3′
*fnbB*	5′-AAGAAGCACCGAAAACTGTG-3′	5′-TCTCTGCAACTGCTGTAACG-3′
*aur*	5′-CAAAAGAGTGATGCGGTCAA-3′	5′-AGGTGCATGAACACCATCAA-3′
*nuc*	5′-GCGATTGATGGTGATACGGTT-3′	5′-AGCCAAGCCTTGACGAACTAAAGC-3′
*crtM*	5′-ATCCAGAACCACCCGTTTTT-3′	5′-GCGATGAAGGTATTGGCATT-3′
*crtN*	5′-GATGAAGCTTTGACGCAACA-3′	5′-TTCGCATGATACGTTTGCTC-3′
*gyrB*	5′-GGTGCTGGGCAAATACAAGT-3′	5′-TCCCACACTAAATGGTGCAA-3′
*RNAIII*	5′-GCACTGAGTCCAAGGAAACTAACTCT-3′	5′-AGCCATCCCAACTTAATAACCATGT-3′
*hld*	5′-TAATTAAGGAAGGAGTGATTTCAATG-3′	5′-TTTTTAGTGAATTTGTTCACTGTGTC-3′
*psmα*	5′-TATCAAAAGCTTAATCGAACAATTC-3′	5′-CCCCTTCAAATAAGATGTTCATATC-3′

### Extracellular protease qualitative assay

Protease agar plates were prepared by adding 1% of casein (Himedia Laboratories) to TSBA, sterilized and poured into petri plates. After solidification, wells were formed in the center of the plates using sterile tips. Cell free culture supernatant collected from control and DD treated MRSA cells (50 µL of each) was added into wells formed and incubated at 37 °C for 16 h. Formation of white zone around well was measured as protease activity^50^.

### Extracellular DNase qualitative assay

DNase test Agar with methyl green (Himedia Laboratories) was prepared, sterilized and poured into petri plates. Control and DD treated MRSA cells were spotted on the DNase agar plates and incubated at 37 °C for 16 h. Zone of clearance around the cells upon green background was observed as DNase activity and measured^51^.

### Exogenous enzymatic treatment

MRSA cells were used to inoculate TSBS to form biofilm on glass slides in the absence and presence of 2 µg/mL of proteinase K (Sigma-aldrich, USA) and 2 units/mL of DNase I (New England Biolabs, USA) separately. After 24 h incubation at 37 °C, glass slides were taken out and washed with sterile PBS which was allowed to air dry. Further, the glass slides were stained and viewed as stated earlier for light microscopy and CLSM analysis^52^.

### Biofilm assay with *agr* mutant strain

Effect of DD on biofilm formation by Newman wild type and *agr* mutant ALC355 strain was examined by MTP assay on polystyrene and glass surfaces. Light microscopic and CLSM analysis was performed as mentioned previously.

### *In vivo* biofilm formation assay

To study the *in vivo* antibiofilm efficacy of DD, *C. elegans*, a simple eukaryotic animal model was used in this study. *C. elegans* were grown in Nematode Growth Medium (NGM) containing *Escherichia coli* OP50 as a laboratory food source. Age synchronized nematodes were exposed to MRSA cells in the absence or presence of DD at BIC and incubated at 20 °C for 24 h. Following the incubation period, the worms were washed thoroughly in M9 buffer to detach any externally adhered MRSA cells and 0.01% Acridine orange was added and incubated for 15 min at room temperature. Then, the worms were then washed with M9 buffer and anesthetized using 20 mM sodium azide and visualized under CLSM. The fluorescence intensity was directly proportional to the rate of bacterial colonization^49^.

### CFU assay

The effect of DD on internal colonization of MRSA within *C. elegans* was assessed by using CFU assay. The nematodes were infected with the test pathogen in the absence and presence of DD at BIC and incubated for 24 h at 20 °C. Following incubation, in order to kill the bacterial cells adhered to the external surface of nematodes, 0.5 μg/ml rifampicin was used for washing the nematodes. 50 μl of PBS along with 0.1% Triton-X 100 was added to the tubes containing worms and were homogenized using micro pestle to release the internalized MRSA bacterial cells. The released bacterial cells were serially diluted and spread plated on Mannitol salt agar plates. The inoculated plates were incubated at 37 °C for 24 h and the total CFU were counted^53^.

### Statistics

All the experiments were carried out in at least three biological replicates with at least two technical triplicates and all the data were presented as mean ± standard deviation. Difference between control and treated groups was statistically analyzed by SPSS 17.0 software package (SPSS Inc., Chicago, IL) using one-way ANOVA followed by Duncan’s post hoc test. The P-value < 0.05 was considered as statistically significant.

## Supplementary information

Supplementary Information
