# Supplementary material for: 5-Dodecanolide interferes with biofilm formation and reduces the virulence of Methicillin-resistant Staphylococcus aureus (MRSA) through up regulation of agr system
Source: Sci Rep. 2019 Sep 24;9:13744. doi: 10.1038/s41598-019-50207-y (PMC6760239; doi:10.1038/s41598-019-50207-y)
Supplement: Supplementary file 1 — Supplementary Information [file 41598_2019_50207_MOESM1_ESM.docx]

**5-Dodecanolide interferes with biofilm formation and reduces the virulence of Methicillin-resistant *Staphylococcus aureus* (MRSA) through up regulation of *agr* system**

Alaguvel Valliammai^1^, Sivasamy Sethupathy^1,2^, Arumugam Priya^1^, Anthonymuthu Selvaraj^1^, James Prabhanand Bhaskar^3^, Venkateswaran Krishnan^3^ and Shunmugiah Karutha Pandian^1^*

^1^Department of Biotechnology, Alagappa University, Science Campus, Karaikudi 630003, Tamil Nadu, India.

^2^School of Chemical Engineering, Yeungnam University, Gyeongsan, 38541, Republic of Korea.

^3^Personal Care Division, ITC LSTC, Bengaluru, India.

*Address correspondence to Shunmugiah Karutha Pandian:

sk_pandian@rediffmail.com; Fax: +91 4565 225202; Tel: +91 4565 225215

**Supplementary Figure S1**: Full image of MTP assay in which red color boxed area is cropped and displayed below the bar graph of Figure 1a.


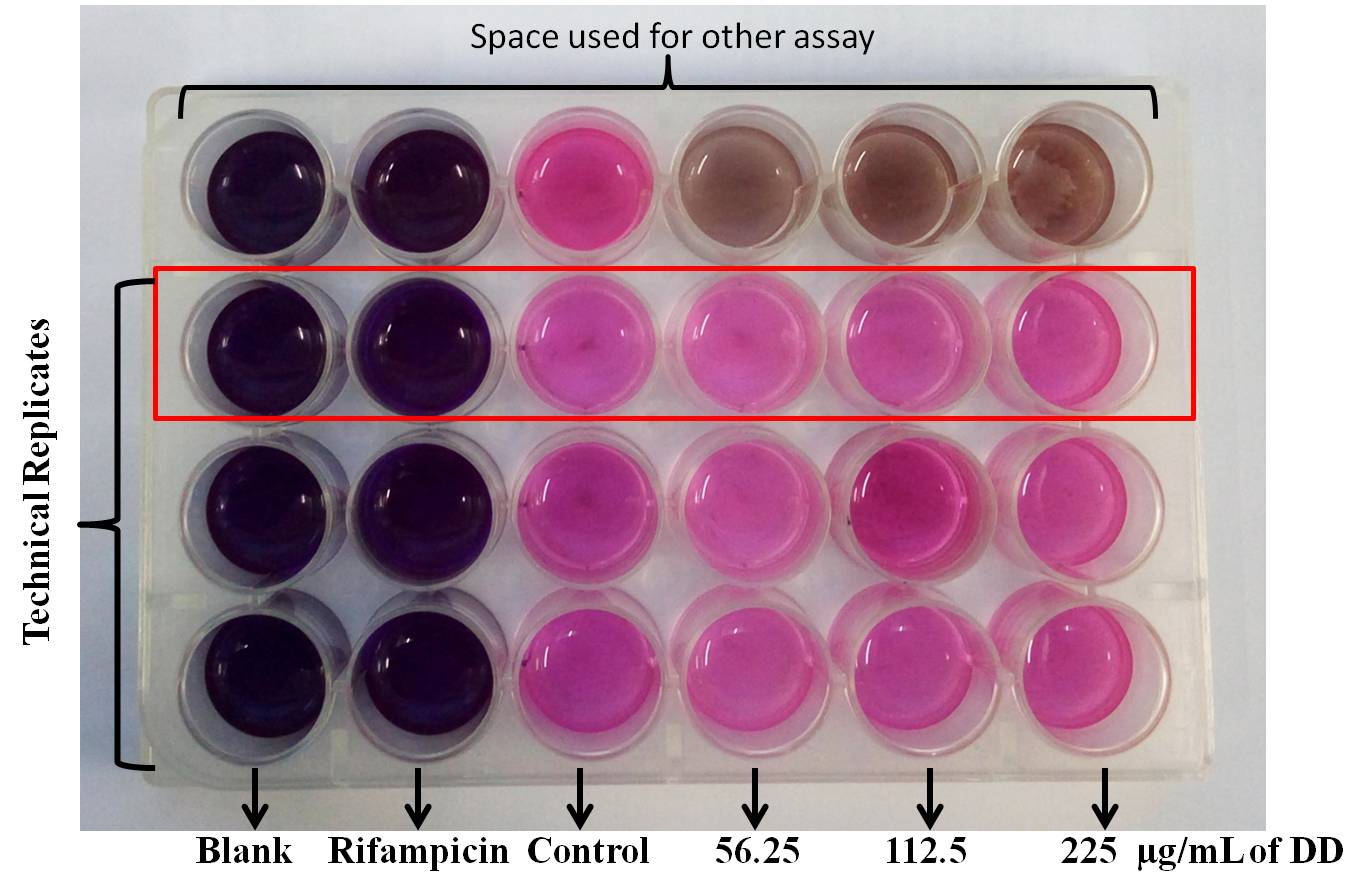


**Supplementary Figure S4**: Full image of alamar blue assay in which red color boxed area is cropped and displayed below the bar graph of Figure 4c.


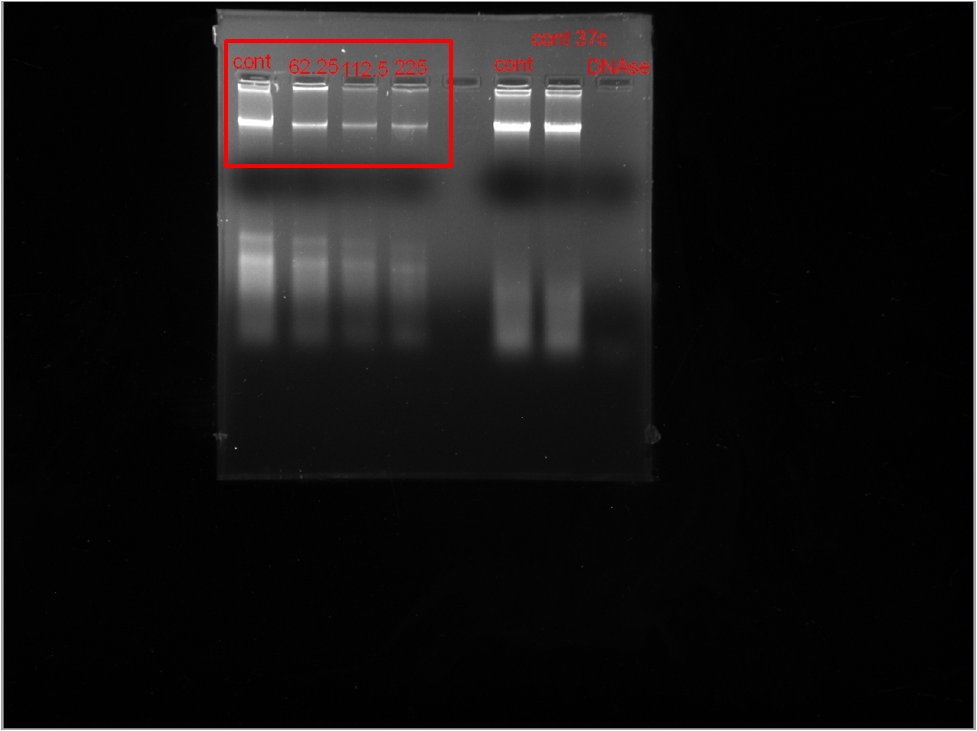


1 2 3 4

Lane 1: Control

Lane 2: 56.25 µg/mL of DD

Lane 3: 112.5 µg/mL of DD

Lane 4: 225 µg/mL of DD

**Supplementary Figure S5**: Uncropped gel image of eDNA in which red color boxed area is cropped and displayed as Figure 5b.


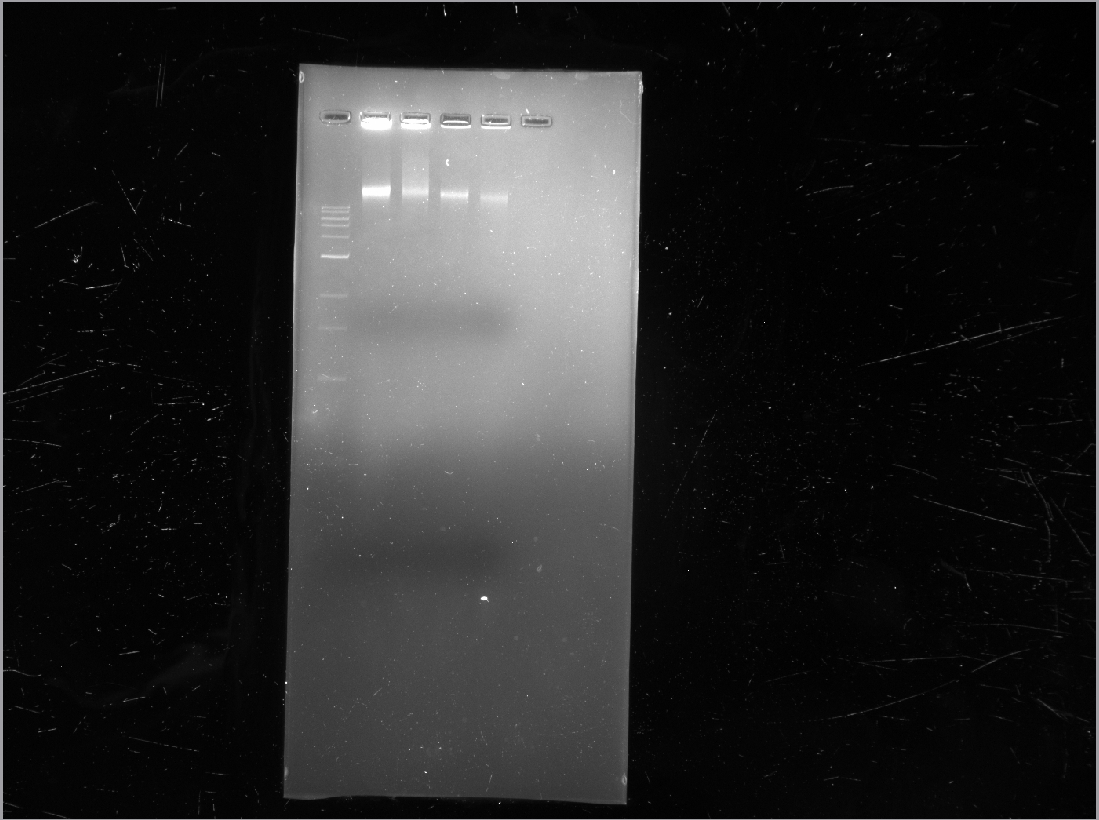


Lane 1: 1 Kb marker

Lane 2: Control

Lane 3: 56.25 µg/mL of DD

Lane 4: 112.5 µg/mL of DD

Lane 5: 225 µg/mL of DD

1 2 3 4 5

**1Kb M C 56.5 112.5 225**

**Supplementary Figure S5b**: Agarose gel electrophoresis of eDNA done with 1 kb marker as per one of the Reviewers suggestion.

.


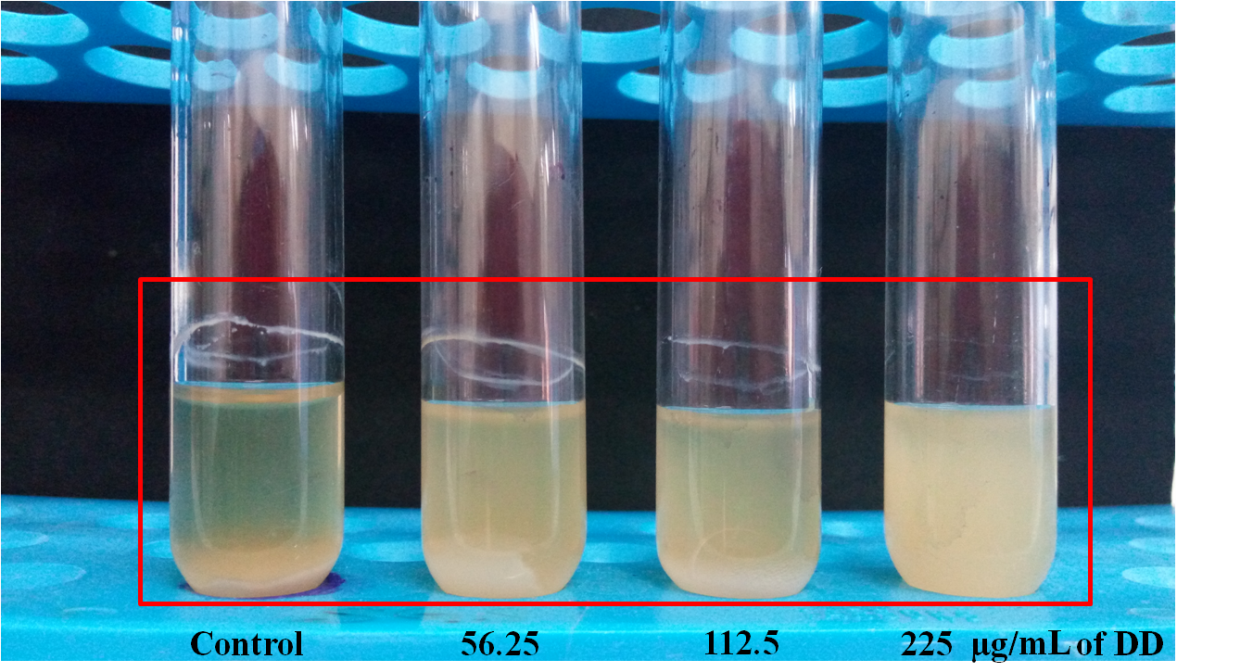


**Supplementary Figure S5**: Full image of autoaggregation assay in which red color boxed area is cropped and displayed as Figure 5d.


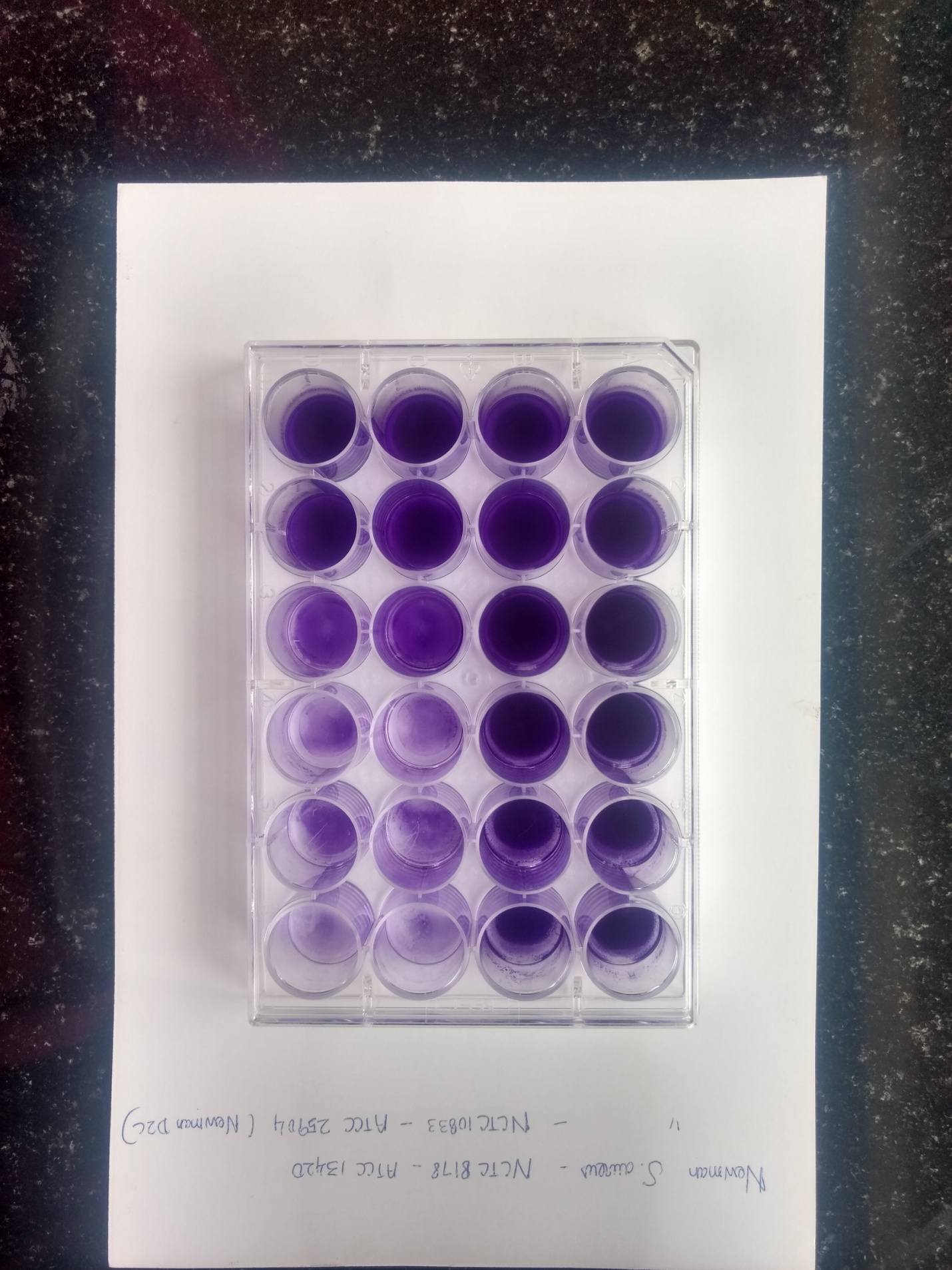


Control

112.5 µg/mL

225 µg/mL

450 µg/mL

900 µg/mL

1000 µg/mL

*agr* mutant

Newman WT

**Supplementary Figure S10**: Uncropped image of MTP assay in which red color boxed area (Wild type) and yellow color (*agr* mutant) boxed area are cropped and displayed below the bar graph of Figure 10a.
